# A Comparison of Wound Complications Following Total Hip Arthroplasty Performed Through the Direct Anterior Versus Direct Lateral Approach

**DOI:** 10.1016/j.artd.2024.101388

**Published:** 2024-05-14

**Authors:** Kurtis D. Carlock, Jacob B. Wilkerson, Jonathan T. Yamaguchi, Navin D. Fernando

**Affiliations:** Department of Orthopaedic Surgery and Sports Medicine, University of Washington, Seattle, WA, USA

**Keywords:** Wound complications, Direct anterior, Direct anterior approach, Direct lateral, Wound healing

## Abstract

**Background:**

Some studies have suggested the risk of wound complications may be higher using the direct anterior (DA) approach to total hip arthroplasty (THA). This study aimed to compare the risk of early postoperative wound complications between the DA and direct lateral (DL) approaches to THA and to determine patient risk factors that may contribute to this problem.

**Methods:**

All patients who underwent primary THA with a single surgeon over a 5-year period were retrospectively reviewed. All patients were treated with either the DA or DL approach. Data collected included patient demographics, surgical approach, and wound status. There was a minimum follow-up of 6 weeks to allow for an adequate assessment of surgical wound healing. Univariate and multivariate analyses were used to compare the 2 approaches.

**Results:**

Five hundred seventy-nine patients (77.6%) who underwent DA approach and 167 patients (22.4%) who underwent DL approach were included. Patients who underwent DL approach had a higher body mass index and a higher rate of diabetes than those treated with the DA approach. Forty patients (6.9%) in the DA cohort and 14 (8.4%) in the DL cohort experienced early wound complications, *P* = .523. After controlling for potential confounding variables, the surgical approach was not an independent risk factor for early postoperative wound complications.

**Conclusions:**

While there have been concerns regarding use of the DA approach in patients with higher body mass index and certain medical comorbidities, the results of this study suggest the choice of surgical approach may have minimal effect on the rate of early postoperative wound complications.

## Introduction

Total hip arthroplasty (THA) has consistently been shown to improve patient health-care-related quality of life, and its application continues to rise each year in the United States and worldwide [[1], [2], [3]]. Additionally, the prevalence of the direct anterior (DA) approach has increased over the last decade, and a recent survey of members of the American Association of Hip and Knee Surgeons demonstrated that a majority of those who responded (56.2%) utilize this approach [4,5]. Supporting its utilization, several studies have suggested that the DA approach may be associated with decreased postoperative pain and a more rapid functional recovery when compared to traditional (posterior or lateral) approaches [[6], [7], [8], [9], [10], [11], [12]]. Furthermore, when compared to posterior-based approaches, some data suggests the DA approach may have a lower rate of postoperative instability, which has been shown to be the most common complication requiring revision in the United States [8,[13], [14], [15], [16]]. With respect to functional outcomes, most studies have suggested equivalence in long-term results based on approach when performed by experienced surgeons [10,11,15,17].

Despite these promising results with respect to stability, pain, and early functional outcome, multiple studies have suggested the DA approach may be associated with a higher rate of superficial wound complications when compared to other approaches [[18], [19], [20], [21], [22], [23]]. This elevated risk is theorized to result from the propensity of the incision to lie near the waist crease and under the abdominal pannus in some obese patients, thereby complicating hygiene and compromising the wound healing environment [19,20,23]. Although some of these superficial wound issues may be limited to delayed wound healing, a subset will develop more extensive complications such as dehiscence, persistent drainage, superficial surgical site infection, or periprosthetic infection. These concerns have likely contributed to the hesitation in adopting this approach and even abandonment by some surgeons [5].

There remains controversy as to whether the DA approach has an intrinsically higher risk of wound complications in comparison to the posterior or direct lateral (DL) approach or if there are specific patient factors that predispose to wound complications irrespective of approach. The purpose of this study was to compare the risk of early postoperative wound complications between the DA and DL approaches to THA and to identify any factors independently associated with early wound issues. We hypothesize that there will be no difference in the rate of wound complications between the 2 approaches in our population.

## Material and methods

Consecutive patients who underwent primary THA performed by a single fellowship-trained orthopaedic surgeon from 2014 through 2019 were retrospectively reviewed using an electronic health record. Data collected included patient demographics, comorbidities, surgical approach, postoperative wound healing, and intervention for any wound complications. Comorbidities analyzed included diabetes mellitus, immunosuppressed status, coronary artery disease, peripheral vascular disease, psoriasis, cirrhosis, hepatitis C viral infection, and rheumatoid arthritis. Patients were considered immunosuppressed if they had been on corticosteroids or disease-modifying anti-rheumatic drugs (DMARDs) or if they carried a diagnosis of an immunodeficiency condition (eg, status post-organ transplant). All patients with at least 6 weeks of postoperative follow-up were included. Six weeks was selected as the required follow-up timepoint to allow for adequate assessment of incision healing; all patients who developed early or persistent wound complications were followed to resolution of their wound healing issue. All patients underwent either a DA or DL approach, and the choice of surgical approach was decided at the discretion of the operating surgeon based on factors including surgical complexity and anticipated exposure needs. The operating surgeon was fellowship-trained in both the DA and DL approaches and routinely employs both approaches in their practice. All patients used a 5-day chlorhexidine gluconate shower preoperatively. An alcohol-based chlorhexidine gluconate surgical prep was used, and 24 hours of perioperative weight-based cefazolin was administered for all cases.

During the study period, 771 patients underwent primary THA. Of these, 25 (3.1%) had less than 6 weeks of postoperative follow-up and were excluded. The remaining 746 patients comprised the study group, of whom 579 (77.6%) underwent the DA approach and 167 (22.4%) underwent the DL approach.

The DA (modified Smith-Peterson) approach involved a skin incision made approximately 2 centimeters lateral and 2 centimeters distal to the anterior superior iliac spine and proceeded distally approximately 10 centimeters oriented toward the lateral femoral condyle. The deep fascia was closed with a running knotless barbed suture, followed by 2-0 absorbable sutures for the deep subcutaneous tissue and subdermal layers. Topical glue was applied to the skin, followed by an occlusive dressing.

The DL (modified Hardinge) approach involved an approximately 10 cm incision centered on the greater trochanter and carried proximally and distally to this landmark. At the conclusion of the THA, the abductor mechanism was closed with a #1 braided absorbable suture, followed by running knotless barbed suture for the iliotibial band, and 2-0 absorbable sutures for the deep subcutaneous and subdermal tissue layers. Topical glue was applied to the skin with an occlusive dressing. The dressing for both DA and DL hips were identical throughout the study period.

Postoperatively, the patients were seen at 2 weeks, 6 weeks, 1 year, and every 5 years thereafter. Patients were considered to have experienced a wound complication if there was any wound dehiscence or drainage at 2 weeks postoperatively, or if there were findings consistent with incisional cellulitis or surgical site infection within 6 weeks of surgery. Assessment of wound complications was completed by the operating surgeon. If delayed wound healing was encountered at the 2-week interval, the patient was prescribed regular dressing changes with weekly follow-up for wound surveillance. If there was suspicion of incisional cellulitis without clinical evidence of deep infection, oral antibiotics were prescribed. If there was suspicion of a superficial surgical site infection or if there was persistent drainage despite dressing changes, patients were treated in the operating room with superficial irrigation and debridement (I&D) as well as wound revision. Any infection that had been suspected to be superficial but was found to track deep to the fascia upon exploration was treated as an acute postoperative prosthetic joint infection (PJI) with modular component exchange.

Patients were divided into 2 groups based on the surgical approach. Statistical analyses were performed to evaluate for differences between patients treated with a DA approach and those treated with a DL approach. Independent samples t tests were used for continuous variables. Chi-squared analyses and Fisher’s exact tests were used for categorical variables. Multivariate logistic regression analysis was performed to evaluate the effect of the surgical approach on the rate of early postoperative wound complications after controlling for potential confounding variables. Covariates assessed in this multivariate analysis included age, sex, body mass index (BMI), and history of diabetes. All statistical calculations were performed using IBM SPSS version 26 (Armonk, NY: IBM Corporation), with significance set at *P* < .05 for all analyses.

## Results

Demographic data for the 2 cohorts is provided in Table 1. Patients who underwent the DL approach had a higher BMI (32.0 ± 7.2 vs 28.3 ± 4.7, *P* < .001), a higher rate of diabetes (21.0% vs 9.8%, *P* < .001), and a higher rate of prior smoking history (47.3% vs 41.3%, *P* = .041) than those who were treated with the DA approach. There was no difference between groups with respect to age, sex, ethnicity, other medical comorbidities, or medication used for postoperative venous thromboembolism prophylaxis. No patients were active tobacco users at the time of surgery. Patients treated with the DL approach had an underlying surgical indication of degenerative hip osteoarthritis in a lower percentage of cases (79.0% vs 87.0%), but a higher rate of avascular necrosis (13.8% vs 10.5%) and primary arthroplasty for hip fracture (7.2% vs 2.1%), *P* = .005.

**Table 1:** Patient demographics and outcomes by surgical approach.

Demographic	Direct anterior (N = 579)	Direct lateral (N = 167)	*P* value
Age (years)	62.2 ± 12.0	60.4 ± 15.1	.114
Body mass index (kg/m^2^)	28.3 ± 4.7	32.0 ± 7.2	<.001^a^
Sex			.093
Male	270 (46.6%)	65 (38.9%)	
Female	309 (53.4%)	102 (61.1%)	
Race			.295
White	496 (85.7%)	135 (80.8%)	
Black	30 (5.2%)	13 (7.8%)	
Hispanic	9 (1.6%)	6 (3.6%)	
Asian/Pacific Islander	31 (5.4%)	10 (6.0%)	
Other	13 (2.2%)	3 (1.8%)	
Prior tobacco use	239 (41.3%)	79 (47.3%)	.041^a^
Comorbidities			
Diabetes	57 (9.8%)	35 (21.0%)	<.001^a^
Immunosuppressed	22 (3.8%)	9 (5.4%)	.380
Coronary artery disease	53 (9.2%)	20 (12.0%)	.301
Peripheral vascular disease	19 (3.3%)	5 (3.0%)	.999
Psoriasis	7 (1.2%)	4 (2.4%)	.277
Hepatitis C	20 (3.4%)	9 (5.4%)	.230
Rheumatoid arthritis	29 (5.0%)	14 (8.4%)	.129
Surgical indication			.005^a^
Osteoarthritis	504 (87.0%)	132 (79.0%)	
Avascular necrosis	61 (10.5%)	23 (13.8%)	
Fracture	14 (2.4%)	12 (7.2%)	
Wound complication			.523
Prolonged dressing changes	17 (2.9%)	4 (2.4%)	
Superficial infection	23 (4.0%)	10 (6.0%)	
Prosthetic joint infection	3 (0.5%)	3 (1.8%)	
Return to OR	15 (2.6%)	6 (3.6%)	.594
Follow-up (weeks)	39.5 ± 41.4	46.5 ± 49.1	.066

a Statistically significant (i.e., *P* < .05).

There were 40 (6.9%) and 14 (8.4%) patients who experienced early wound complications in the DA and DL cohorts, respectively. The rate of wound complications did not differ between groups, *P* = .523. Among the 40 patients in the DA cohort who developed early wound issues, 17 (2.9%) experienced delayed wound healing that resolved with prolonged dressing changes and wound care, 8 (1.4%) developed incisional cellulitis that resolved with oral antibiotics, and 15 (2.6%) developed a surgical site infection for which they returned to the operating room. Of those who returned to the operating room, 12 (2.1%) underwent isolated superficial I&D with wound revision, while 3 (0.5%) had findings concerning acute PJI and were treated with debridement, antibiotics, and implant retention with the exchange of modular components. Among the 14 patients in the DL cohort who developed early wound issues, 5 (3.0%) experienced delayed wound healing that resolved with prolonged dressing changes and wound care, 3 (1.8%) developed incisional cellulitis that resolved with oral antibiotics, and 6 (3.6%) developed a surgical site infection for which they returned to the operating room. Of those who returned to the OR, 3 (1.8%) underwent isolated superficial I&D with wound revision, while 3 (1.8%) had findings concerning acute PJI and were treated with debridement, antibiotics, and implant retention with exchange of modular components. There was no statistical difference between groups with respect to the rate of return to OR for early wound complications (*P* = .594).

A comparison of demographic data between patients who developed an early wound complication and those who did not is provided in Table 2. Patients with early wound complications were older (65.2 ± 11.3 years vs 61.5 ± 12.8 years, *P* = .041), had a higher BMI (32.8 ± 5.7 vs 28.8 ± 5.4, *P* < .001), and were more likely to be diabetic (22.2% vs 11.6%, *P* = .030). There was no association between the development of early wound complications and sex, ethnicity, smoking history, immunosuppression or other medical comorbidities, surgical indication, or surgical approach.

**Table 2:** Comparison between patients who did and did not develop an early wound complication.

Demographic	No wound complication (N = 692)	Wound complication (N = 54)	*P* value
Age (years)	61.5 ± 12.8	65.2 ± 11.3	.041^a^
Body mass index (kg/m^2^)	28.8 ± 5.4	32.8 ± 5.7	<.001^a^
Sex			.088
Male	317 (45.8%)	18 (33.3%)	
Female	375 (54.2%)	36 (66.7%)	
Race			.790
White	582 (84.1%)	49 (90.7%)	
Black	41 (5.9%)	2 (3.7%)	
Hispanic	14 (2.0%)	1 (1.9%)	
Asian/Pacific Islander	40 (5.8%)	1 (1.9%)	
Other	15 (2.2%)	1 (1.9%)	
Prior tobacco use	296 (42.8%)	22 (40.7%)	.778
Comorbidities			
Diabetes	80 (11.6%)	12 (22.2%)	.030^a^
Immunosuppressed	27 (3.9%)	4 (7.4%)	.272
Coronary artery disease	65 (9.4%)	8 (14.8%)	.229
Peripheral vascular disease	21 (3.0%)	3 (5.6%)	.408
Psoriasis	10 (1.4%)	1 (1.9%)	.565
Hepatitis C	27 (3.9%)	2 (3.7%)	.780
Rheumatoid arthritis	38 (5.5%)	5 (9.3%)	.229
Postoperative anticoagulation			.315
Aspirin	591 (85.4%)	42 (77.8%)	
Enoxaparin	7 (1.0%)	2 (3.7%)	
Warfarin	47 (6.8%)	6 (11.1%)	
Rivaroxaban	19 (2.7%)	2 (3.7%)	
Apixaban	24 (3.5%)	2 (3.7%)	
Clopidogrel	3 (0.4%)	0 (0%)	
Heparin	1 (0.1%)	0 (0%)	
Surgical indication			.150
Osteoarthritis	591 (85.4%)	45 (83.3%)	
Avascular necrosis	75 (10.8%)	9 (16.7%)	
Fracture	26 (3.8%)	0 (0.0%)	
Surgical approach			.611
Direct anterior	539 (77.9%)	40 (74.1%)	
Direct lateral	153 (22.1%)	14 (25.9%)	
Follow-up (weeks)	49.7 ± 42.6	45.5 ± 51.5	.434

a Statistically significant (i.e., *P* < .05).

Following control of potential confounding variables, surgical approach demonstrated no association with the development of early postoperative wound complications (*P* = .155). Factors that were independently associated with wound complications were increasing age (odds ratio = 1.033 [1.005-1.061], *P* = .019) and increasing BMI (odds ratio = 1.149 [1.087-1.215], *P* < .001). The complete results for all covariates included in the logistic regression analysis are included in Table 3.

**Table 3:** Multivariate analysis examining possible risk factors for early wound complication.

Covariate	Odds ratio	95% Confidence interval	*P* value
Surgical approach	0.586	0.280-1.225	.155
Age	1.033	1.005-1.061	.019^a^
Sex	0.613	0.336-1.120	.111
Body mass index	1.149	1.087-1.215	<.001^a^
History of diabetes	1.617	0.788-3.317	.190

a Statistically significant (i.e., *P* < .05).

A post-hoc power analysis demonstrated a calculated power of 10.8% to detect a difference in the rate of wound complications between the 2 cohorts within the specified significance level of 0.05.

## Discussion

Our study identified elevated BMI and increasing age as factors independently associated with the development of early postoperative wound complications in THA patients. The surgical approach used (DA vs DL) was not a risk factor for early wound complications in our cohort, although our study may have been underpowered to identify such a difference. These findings suggest that intrinsic patient factors may have a greater impact on wound healing complications than the choice of surgical approach.

Multiple prior studies have directly compared the DA and DL approaches for THA, with the current body of literature suggesting no obvious inherent superiority of one approach over another (particularly with respect to long-term outcomes). Several randomized controlled trials (RCTs) have examined the functional outcome differences between the 2 approaches; although several have shown superior early functional outcome measures in patients treated with the DA approach, others have shown early equivalence [[9], [10], [11],^17^,^24^]. Regardless, all RCTs published to date of which we are aware demonstrate no clinically relevant difference between the 2 approaches by 1 year postoperatively [[9], [10], [11], [17], [24]]. Additionally, a 2019 RCT found no variation in component alignment between the 2 groups when the same target implant position was sought [25]. With respect to postoperative complications, multiple RCTs and appropriately powered retrospective cohort studies have suggested a greater risk of complications such as infection, wound issues, fracture, component loosening, and nerve injury with the DA approach when compared to the DL approach [9,16,17,20,21,24,26]. Other high-quality studies, however, have shown no difference in complication profile between the 2 approaches, particularly past the learning curve [10,24,[27], [28], [29]].

When evaluating wound complications and surgical site infections specifically, the current literature also remains unclear. A well-powered retrospective comparative study by Aggarwal et al found the DA approach to have a significantly higher rate of prolonged wound drainage, superficial infection, and deep infection when compared to the DL approach [21]. This is in contrast to well-powered cohort studies by Hart et al and Ilchmann et al that found no difference in the rates of wound dehiscence, superficial infection, and PJI between the 2 approaches [28,29]. Furthermore, a 2018 RCT found a 2% rate of wound healing issues that resolved with nonoperative care following THA via both DA and DL approaches [9].

While some studies have suggested the DA approach may be associated with an elevated risk of postoperative complications, the ability to analyze risk propensity is convoluted by the repeated finding that the DA approach has an established learning curve during which the rate of several complications is higher than after surgeons have gained further experience in the technique [18,21,26,30]. The rate of wound complications following DA THA, however, has been shown to be unrelated to the number of prior cases performed by the operating surgeon, suggesting that such issues are unrelated to the surgical learning curve [19,31].

Prior studies have identified multiple risk factors for wound complications following THA performed utilizing the DA approach. A retrospective case-control study by Jahng et al identified a BMI greater than 40 kg/m^2^ and a history of diabetes to be independent risk factors for wound complications, and these were associated with a 5-fold and 13-fold increase in risk for reoperation related to wound complications, respectively [32]. The effect of obesity on wound complications has been well studied in patients undergoing THA utilizing a DA approach. Obesity has been associated with an increased rate of delayed wound healing, wound dehiscence, superficial surgical site infection, PJI, and the overall rate of reoperation [19,20,23,28,33].

Additionally, tobacco use has established consequences on wound healing and has been associated with wound healing issues following DA THA [34]. Increasing age also has known impacts on wound healing ability, although the effect of age on wound healing in the THA population, specifically, remains poorly studied [35]. Lastly, female sex has also been identified as a risk factor for wound complications following DA THA [19]. Our study is in alignment with prior literature in its identification of increasing BMI and age as risk factors for early wound complications in patients undergoing primary THA, although female sex and a history of smoking were not independent risk factors in this study.

As more surgeons have become proficient with the DA approach for THA, there has been a push for further innovation to address the potential shortcomings of the technique. Recently, some surgeons have trialed the use of a bikini-style incision as opposed to the more traditional longitudinal incision. Early results of studies examining this technique have shown conflicting results, with some demonstrating the bikini-style incision to be associated with a lower rate of wound complications while others have shown no difference from the longitudinal incision [36,37]. Additional research is needed to assess the safety and complication profile associated with this technique.

This study has several notable limitations. It is inherently limited in its design as a retrospective, single surgeon cohort study, which introduces the risk of selection bias. This was illustrated by the unequal demographic distribution of patients in our study population, with a trend toward higher BMI, older age, and smoking history in those patients treated with the DL approach. We attempted to control for this through our use of multivariate analyses, but a prospective and randomized study would have superior methodology. Our results should be interpreted in the setting of this known risk for selection bias. Additionally, as suggested by our post-hoc power analysis, the possibility of an underlying type 2 error has not been excluded, and it is important to acknowledge that our results do not demonstrate clear equivalence of the DA and DL approaches. Our study also provides an isolated look at early postoperative wound complications. It is possible that some long-term risks inherent to each approach, such as a later presentation of PJI or recurrence, were not captured in our analysis; as such, our results should be interpreted solely with respect to the early postoperative period. Lastly, a subset of medical comorbidities and their impact on the development of wound complications was examined; it is possible other comorbidities not included in our analysis had an impact on wound healing that was not detected.

There are several strengths to our study that are also of note. First, this study represents a consecutive case-control series performed by a single surgeon using identical closure technique, which intrinsically should minimize variables seen in multisurgeon studies utilizing various techniques and dressings. Secondly, this study was performed at a tertiary safety-net academic institution and included a high volume of patients with high-morbidity profiles. Despite this morbidity bias, complications rates were similar among treatment arms, and independent risk factors for wound complications regardless of surgical approach were identified. Lastly, this study group represents those patients treated within the first 5 years of the participating surgeon’s practice postfellowship. Given training trends in adult joint reconstruction fellowships, with a growing emphasis toward expertise in DA approach surgery, the results of this study support the utilization of DA approach for eligible patients throughout the learning curve. Patients with a higher BMI, diabetes, and advanced age, however, should be counseled regarding a potentially increased risk of would complication, irrespective of the choice of surgical approach.

## Conclusions

The results of our study suggest that the DA and DL approaches for THA may be similar with respect to the rate of early postoperative wound complications, although better powered and/or prospective studies are needed to make definitive conclusions. Increasing BMI and increasing age were identified as independent risk factors for the development of early wound issues, regardless of the surgical approach utilized. Patients with these risk factors should be counseled appropriately prior to undergoing THA.

## Conflicts of interest

The authors declare there are no conflicts of interest.

For full disclosure statements refer to https://doi.org/10.1016/j.artd.2024.101388.

## Statement of IRB approval

This study was approved by the University of Washington School of Medicine Institutional Review Board.

## CRediT authorship contribution statement

**Kurtis D. Carlock:** Conceptualization, Data curation, Formal analysis, Methodology, Writing – original draft, Writing – review & editing. **Jacob B. Wilkerson:** Conceptualization, Data curation, Formal analysis, Writing – original draft. **Jonathan T. Yamaguchi:** Formal analysis, Writing – original draft, Writing – review & editing. **Navin D. Fernando:** Conceptualization, Supervision, Writing – original draft, Writing – review & editing.

## Appendix A Supplementary data

Conflict of Interest Statement for Carlock

Conflict of Interest Statement for Fernando

Conflict of Interest Statement for Wilkerson

Conflict of Interest Statement for Yamaguchi

## References

[1] Kurtz S., Ong K., Lau E., Mowat F., Halpern M. (2007). Projections of primary and revision hip and knee arthroplasty in the United States from 2005 to 2030. J Bone Joint Surg Am.

[2] Ackerman I.N., Bohensky M.A., Zomer E., Tacey M., Gorelik A., Brand C.A. (2019). The projected burden of primary total knee and hip replacement for osteoarthritis in Australia to the year 2030. BMC Musculoskelet Disord.

[3] Patel A., Pavlou G., Mújica-Mota R.E., Toms A.D. (2015). The epidemiology of revision total knee and hip arthroplasty in England and Wales: a comparative analysis with projections for the United States. A study using the National Joint Registry dataset. Bone Joint Lett J.

[4] Abdel M.P., Berry D.J. (2019). Current practice trends in primary hip and knee arthroplasties among members of the American association of hip and knee surgeons: a long-term update. J Arthroplasty.

[5] Woolson S.T. (2020). A survey of Hip Society surgeons concerning the direct anterior approach total hip arthroplasty. Bone Joint Lett J.

[6] Wang Z., Bao H.-W., Hou J.-Z. (2019). Direct anterior versus lateral approaches for clinical outcomes after total hip arthroplasty: a meta-analysis. J Orthop Surg Res.

[7] Nakata K., Nishikawa M., Yamamoto K., Hirota S., Yoshikawa H. (2009). A clinical comparative study of the direct anterior with mini-posterior approach: two consecutive series. J Arthroplasty.

[8] Barrett W.P., Turner S.E., Leopold J.P. (2013). Prospective randomized study of direct anterior vs postero-lateral approach for total hip arthroplasty. J Arthroplasty.

[9] Brismar B.H., Hallert O., Tedhamre A., Lindgren J.U. (2018). Early gain in pain reduction and hip function, but more complications following the direct anterior minimally invasive approach for total hip arthroplasty: a randomized trial of 100 patients with 5 years of follow up. Acta Orthop.

[10] Parvizi J., Restrepo C., Maltenfort M.G. (2016). Total hip arthroplasty performed through direct anterior approach provides superior early outcome: results of a randomized, prospective study. Orthop Clin North Am.

[11] Restrepo C., Parvizi J., Pour A.E., Hozack W.J. (2010). Prospective randomized study of two surgical approaches for total hip arthroplasty. J Arthroplasty.

[12] Mirza A.J., Lombardi A.V., Morris M.J., Berend K.R. (2014). A mini-anterior approach to the hip for total joint replacement: optimising results: improving hip joint replacement outcomes. Bone Joint Lett J.

[13] Bozic K.J., Kurtz S.M., Lau E., Ong K., Vail T.P., Berry D.J. (2009). The epidemiology of revision total hip arthroplasty in the United States. J Bone Joint Surg Am.

[14] Sheth D., Cafri G., Inacio M.C.S., Paxton E.W., Namba R.S. (2015). Anterior and anterolateral approaches for THA are associated with lower dislocation risk without higher revision risk. Clin Orthop Relat Res.

[15] Barrett W.P., Turner S.E., Murphy J.A., Flener J.L., Alton T.B. (2019). Prospective, randomized study of direct anterior approach vs posterolateral approach total hip arthroplasty: a concise 5-year follow-up evaluation. J Arthroplasty.

[16] Fleischman A.N., Tarabichi M., Magner Z., Parvizi J., Rothman R.H. (2019). Mechanical complications following total hip arthroplasty based on surgical approach: a large, single-institution cohort study. J Arthroplasty.

[17] Mjaaland K.E., Kivle K., Svenningsen S., Nordsletten L. (2019). Do postoperative results differ in a randomized trial between a direct anterior and a direct lateral approach in THA?. Clin Orthop Relat Res.

[18] Flevas D.A., Tsantes A.G., Mavrogenis A.F. (2020). Direct anterior approach total hip arthroplasty revisited. JBJS Rev.

[19] Watts C.D., Houdek M.T., Wagner E.R., Sculco P.K., Chalmers B.P., Taunton M.J. (2015). High risk of wound complications following direct anterior total hip arthroplasty in obese patients. J Arthroplasty.

[20] Christensen C.P., Karthikeyan T., Jacobs C.A. (2014). Greater prevalence of wound complications requiring reoperation with direct anterior approach total hip arthroplasty. J Arthroplasty.

[21] Aggarwal V.K., Elbuluk A., Dundon J., Herrero C., Hernandez C., Vigdorchik J.M. (2019). Surgical approach significantly affects the complication rates associated with total hip arthroplasty. Bone Joint Lett J.

[22] Herndon C.L., Drummond N., Sarpong N.O., Cooper H.J., Shah R.P., Geller J.A. (2020). Direct anterior versus mini-anterolateral approach for primary total hip arthroplasty: early postoperative outcomes and complications. Arthroplast Today.

[23] Purcell R.L., Parks N.L., Gargiulo J.M., Hamilton W.G. (2016). Severely obese patients have a higher risk of infection after direct anterior approach total hip arthroplasty. J Arthroplasty.

[24] Huang X.-T., Liu D.-G., Jia B., Xu Y.-X. (2021). Comparisons between direct anterior approach and lateral approach for primary total hip arthroplasty in postoperative orthopaedic complications: a systematic review and meta-analysis. Orthop Surg.

[25] Brun O.-C.L., Sund H.N., Nordsletten L., Röhrl S.M., Mjaaland K.E. (2019). Component placement in direct lateral vs minimally invasive anterior approach in total hip arthroplasty: radiographic outcomes from a prospective randomized controlled trial. J Arthroplasty.

[26] Pincus D., Jenkinson R., Paterson M., Leroux T., Ravi B. (2020). Association between surgical approach and major surgical complications in patients undergoing total hip arthroplasty. JAMA.

[27] Chen A.F., Chen C.-L., Low S., Lin W.-M., Chinnakkannu K., Orozco F.R. (2016). Higher acetabular anteversion in direct anterior total hip arthroplasty: a retrospective case-control study. HSS J.

[28] Ilchmann T., Zimmerli W., Bolliger L., Graber P., Clauss M. (2016). Risk of infection in primary, elective total hip arthroplasty with direct anterior approach or lateral transgluteal approach: a prospective cohort study of 1104 hips. BMC Musculoskelet Disord.

[29] Hart A., Wyles C.C., Abdel M.P., Perry K.I., Pagnano M.W., Taunton M.J. (2019). Thirty-day major and minor complications following total hip arthroplasty-A comparison of the direct anterior, lateral, and posterior approaches. J Arthroplasty.

[30] Jewett B.A., Collis D.K. (2011). High complication rate with anterior total hip arthroplasties on a fracture table. Clin Orthop Relat Res.

[31] Hallert O., Li Y., Brismar H., Lindgren U. (2012). The direct anterior approach: initial experience of a minimally invasive technique for total hip arthroplasty. J Orthop Surg Res.

[32] Jahng K.H., Bas M.A., Rodriguez J.A., Cooper H.J. (2016). Risk factors for wound complications after direct anterior approach hip arthroplasty. J Arthroplasty.

[33] Antoniadis A., Dimitriou D., Flury A., Wiedmer G., Hasler J., Helmy N. (2018). Is direct anterior approach a credible option for severely obese patients undergoing total hip arthroplasty? A matched-control, retrospective, clinical study. J Arthroplasty.

[34] Pachter C.S., Garfinkel J.H., Cochrane N.H., Romness D.W., Gladnick B.P. (2020). Smoking is a risk factor for wound complications after direct anterior hip arthroplasty with mesh tape closure. Adv Skin Wound Care.

[35] Bonifant H., Holloway S. (2019). A review of the effects of ageing on skin integrity and wound healing. Br J Community Nurs.

[36] Manrique J., Paskey T., Tarabichi M., Restrepo C., Foltz C., Hozack W.J. (2019). Total hip arthroplasty through the direct anterior approach using a bikini incision can Be safely performed in obese patients. J Arthroplasty.

[37] Wang Q., Yue Y., Yang Z., Chen L., Li Q., Kang P. (2021). Comparison of postoperative outcomes between traditional longitudinal incision and bikini incision in total hip arthroplasty via direct anterior approach: a randomized controlled trial. J Arthroplasty.

