# Supplementary material for: A Comparison of Wound Complications Following Total Hip Arthroplasty Performed Through the Direct Anterior Versus Direct Lateral Approach
Source: Arthroplast Today. 2024 May 14;27:101388. doi: 10.1016/j.artd.2024.101388 (PMC11106518; doi:10.1016/j.artd.2024.101388)
Supplement: Conflict of Interest Statement for Carlock [file mmc1.docx]

# INDIVIDUAL CONFLICT OF INTEREST STATEMENT

***American Association of Hip and Knee Surgeons***

(Adopted from the American Academy of Orthopaedic Surgeons disclosure statement)

The following form **must be filled out completely and submitted by each author (example, 6 authors, 6 forms).**

**All items require a response. If there is no relevant disclosure for a given item, enter "*None*.”**

No Difference in the Incidence of Wound Complications following Total Hip Arthroplasty Performed Through the Direct Anterior Versus Direct Lateral Approach: A Prospective Cohort Study

**Manuscript Title**

1. Royalties from a company or supplier (The following conflicts were disclosed)

*None*

2. Speakers bureau/paid presentations for a company or supplier (The following conflicts were disclosed)

*None*

3A. Paid employee for a company or supplier (The following conflicts were disclosed)

*None*

3B. Paid consultant for a company or supplier (The following conflicts were disclosed)

*None*

3C. Unpaid consultants for a company or supplier (The following conflicts were disclosed)

*None*

4. Stock or stock options in a company or supplier (The following conflicts were disclosed)

*None*

5. Research support from a company or supplier as a Principal Investigator (The following conflicts were disclosed)

*None*

6. Other financial or material support from a company or supplier (The following conflicts were disclosed)

*None*

7. Royalties, financial or material support from publishers (The following conflicts were disclosed)

*None*

8. Medical/Orthopaedic publications editorial/governing board (The following conflicts were disclosed)

*None*

9. Board member/committee appointments for a society (The following conflicts were disclosed)

*None*

**Each author must sign AND print or type his/her name, date and submit a separate form**

In addition, one BLINDED Conflict of Interest form (no author names used) should be submitted per manuscript with all author disclosures.

Kurtis D. Carlock Kurtis D. Carlock 5/25/23

Author Name (Print or Type) Author Signature Date
